# Interplay between craniofacial stem cells and immune stimulus

**DOI:** 10.1186/s13287-017-0607-1

**Published:** 2017-06-24

**Authors:** Ruili Yang, Tingting Yu, Yanheng Zhou

**Affiliations:** Center for Craniofacial Stem Cell Research and Regeneration, Department of Orthodontics, Peking University School and Hospital of Stomatology, National Engineering Laboratory for Digital and Material Technology of Stomatology, Beijing Key Laboratory of Digital Stomatology, #22 Zhongguancun South Avenue, Beijing, 100081 China

**Keywords:** Mesenchymal stem cells, Craniofacial tissue, Immunity, Osteogenesis, Regenerative medicine

## Abstract

Craniofacial mesenchymal stem cells (MSCs), isolated from an abundant and accessible source of craniofacial tissues, possess self-renewal and multilineage differentiation potential. It has been reported that craniofacial MSCs show elevated proliferation and regeneration capacities compared to bone marrow mesenchymal stem cells (BMMSCs). Furthermore, the immunomodulatory property has generated an emerging multidisciplinary research field that translates MSC-based therapies to the clinic for the treatment of inflammatory and autoimmune diseases. Due to tremendous unmet clinical needs, it was extensively investigated how craniofacial MSCs impose their therapeutic effects, especially by interacting with immune cells. Mechanically, MSCs take advantage of a variety of pathways to regulate immune cells, including paracrine signaling such as transforming growth factor (TGF)-β and hepatocyte growth factor (HGF) pathways, and cell-cell contact Fas/FasL signaling-induced apoptosis. In return, immune cells attenuate MSC function by secreting inflammatory cytokines such as tumor necrosis factor (TNF)-α and interleukin (IL)-1β. This perspective review critically discusses the interaction of craniofacial MSCs with the immune milieu, as well as the underlying molecular mechanism contributing to the future improved therapeutic effects of craniofacial MSCs.

## Background

Mesenchymal stem cells (MSCs), first identified by Friedenstein as colony-forming unit-fibroblasts (CFU-F) [1], are a heterogeneous population that possesses multilineage differentiation potential to bone, fat, and cartilage. In addition to the regenerative potential, emerging evidence has shown that MSCs can regulate the immune status [2] by a sophisticated molecular network. Therefore, the immunomodulatory properties of MSCs and their underlying mechanisms have been extensively studied.

MSCs have been identified from a plethora of bone marrow, skin, adipose tissue, tendon, and craniofacial tissue. The oral cavity has a higher frequency of exposure to the immune and inflammatory environment than skeletal tissues [3, 4], which make craniofacial MSCs a unique population in immunomodulation. In this article, we discuss recent discoveries concerning the properties of craniofacial MSCs, especially the interplay between craniofacial MSCs and the immune microenvironment.

## Mesenchymal stem cells from craniofacial tissues

Currently, various populations of MSCs have been identified in craniofacial tissues, including dental pulp stem cells (DPSCs) [5], stem cells of human exfoliated deciduous teeth (SHED) [6], periodontal ligament stem cells (PDLSCs) [7], dental follicle precursor cells, stem cells from apical papilla, mesenchymal stem cells derived from jaw bone [8], and stem cells derived from gingiva (GMSCs) [9]. Similar to bone marrow MSCs (BMMSCs), craniofacial MSCs express a panel of mesenchymal stem cell markers such as CD73, CD29, CD146, CD105, CD90, and CD44, but are negative for hematopoietic makers CD34 and CD45. Like BMMSCs, craniofacial MSCs possess self-renewal and osteogenic, adipogenic, and chondrogenic differentiation potential. Related to their origin, these craniofacial MSCs harbor the capacity to uniquely differentiate into dentin, cementum, and periodontal ligament-like tissues of the craniofacial region [8, 9]. In terms of immunomodulatory potential, these MSCs showed similar/higher capacity than their skeletal counterparts.

## The origin of craniofacial MSCs

Distinct from BMMSCs, craniofacial MSCs are derived from the neural crest and mesoderm during development. Cranial neural crest cells (CNCCs) give rise to mesenchymal structures, such as neural tissues, cartilage, bone, and teeth in the craniofacial region [10]. Studies have shown that the progenitor cells from the oral mucosa lamina propria and gingiva are possibly derived from CNCCs [11]. Moreover, approximately 90% of GMSCs are derived from CNCCs, along with 10% derived from the mesoderm. GMSCs derived from CNCCs show an increased capacity to differentiate into neural cells and chondrocytes, as well as to modulate immune cell function, compared to GMSCs derived from the mesoderm [11]. CNCCs possess an elevated capacity for both self-renewal and generation of multiple kinds of progenitors under appropriate conditions [12], especially in regard to the generation of neuronal and glial cells [13], which may contribute to the unique properties of craniofacial MSCs, as distinct from BMMSCs.

## Immunomodulatory properties of craniofacial MSCs

**Fig. 1:**
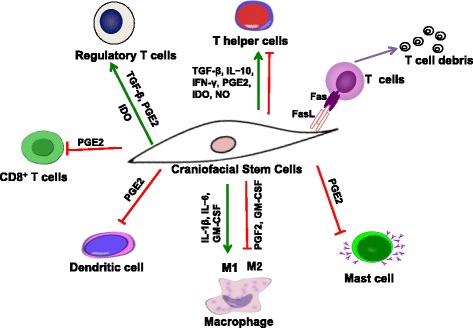
Immunomodulatory properties of craniofacial MSCs. Craniofacial MSCs target several subsets of innate and adaptive immune cells, including helper T-lymphocytes (Ths), CD8^+^ T cells, dentritic cells (DCs), macrophages, mast cells, and regulatory T-lymphocytes (Treg). These effects may be mediated by soluble factors secreted by MSCs, such as prostaglandin E2 (*PGE2*), transforming growth factor-β1 (*TGF-β*), nitric oxide (*NO*), and indoleamine 2,3-dioxygenase (*IDO*), or in a cell-cell contact-dependent manner, inducing T-cell apoptosis through the Fas/FasL pathway. *GM-CSF* granulocyte-macrophage colony stimulating factor, *IFN* interferon, *IL* interleukin

Accumulating evidence has shown that systemic infusion of BMMSCs induced significant immunomodulatory effects in inflammatory and autoimmune diseases. These effects include inhibiting proliferation of immune cells, such as dendritic cells (DCs), T cells, and natural killer (NK) cells, promoting Foxp3^+^ regulatory T (Treg) cells in both function and proliferation [14–16]. The immunomodulatory properties of MSCs are further associated with cell-cell contact *via *Fas/FasL apoptosis pathways or the production of a variety of cytokines and chemokines, such as interleukin (IL)-10, nitric oxide (NO), tumor necrosis factor-inducible gene 6 (TSG-6), prostaglandin E2 (PGE2), and transforming growth factor (TGF)-β [14, 17–19]. These ‘immunologically privileged’ craniofacial MSCs possess immunomodulatory properties that are comparable or superior to those of bone marrow counterparts *via* different mechanisms (Fig. 1), which make them promising alternative cell sources for immunotherapy.

### Dental pulp stem cells

Since Gronthos et al. first identified DPSCs in 2000, experimental and clinical evidence has shown that DPSCs are able to regenerate a dentin/pulp-like complex and bone tissue, and display strong immunosuppressive capacity [5]. DPSCs inhibit proliferation of T cells more effectively than BMMSCs [20]. Moreover, DPSCs inhibit peripheral blood mononuclear cell (PBMC) proliferation in an allogeneic mixed lymphocyte reaction (MLR) via secreting soluble factors such as TGF-β, hepatocyte growth factor (HGF), and indoleamine 2,3-dioxygenase (IDO) [21]. This immunosuppressive activity makes DPSCs better candidates for suppression of T cell-mediated reactions in allogeneic bone marrow transplantation. In addition, DPSCs induced activated T-cell apoptosis in vitro via the Fas/FasL pathway and ameliorated inflammatory injuries when systemically infused into a murine colitis model.

### Gingiva-derived MSCs

Zhang et al. isolated and identified a distinct population of MSCs from gingiva (GMSCs) which can be conveniently acquired from discarded gingiva samples [9]. In addition to higher proliferation and regeneration capacities than BMMSCs, the immunomodulatory abilities of GMSCs have attracted extensive attention [9]. Several studies have investigated the immunomodulatory effects of GMSCs and their interplay with innate and adaptive immune cells. GMSCs display suppressive effects on proliferation and activation of PBMCs in a cell-cell contact-independent manner, apparently mediated via IDO, whereas interferon (IFN)-γ or co-culture with activated T cells leads to upregulation of IDO [22]. Similar immunosuppressive effects on PBMCs stimulated by allogeneic lymphocytes in MLRs have been reported [23]. In addition, GMSCs inhibit Th17 cell differentiation and promote Treg cell expansion [9, 23, 24]. The immunomodulation on T cells make GMSCs a promising alternative source of cells for treating inflammation and immune diseases. Systemic infusion of GMSCs has been shown to attenuate the dextran sulfate sodium (DSS)-induced murine colitis phenotype, producing beneficiary effects such as reversing body weight loss, improving overall colitis score, and rescuing intestinal architecture. Mechanically, GMSC treatment reduced infiltration of CD4^+^ IFN-γ^+^ (Th1) and CD4^+^ IL-17^+^ (Th17) cells with reduction of the inflammatory cytokines IL-17, IL-6, and IFN-γ, whereas it increased recruitment of Treg cells with increased IL-10 [9]. In addition, GMSC infusion exhibited remarkable immune tolerance and promoted the survival of skin allografts through increased infiltration of Tregs [23].

Interestingly, GMSCs also exhibit immunomodulatory effects on innate immune cells, particularly DCs, macrophages, and mast cells [24, 25]. For instance, GMSCs were reported to inhibit the maturation and activation of DCs via production of PGE2, which contributes to the therapeutic effect of GMSCs on hapten (oxazolone)-induced murine contact hypersensitivity (CHS). Moreover, GMSCs also inhibit infiltration of CD8^+^ T cells, Th17, and mast cells, decrease inflammatory cytokines, and induce a reciprocal increased infiltration of Treg cells via the cyclooxygenase 2 (COX2)/PGE2 axis [24]. Similar to BMMSCs, GMSCs were shown to be capable of polarizing macrophages into the M2 phenotype, which is considered to be anti-inflammatory, via enhanced secretion of IL-6 and granulocyte-macrophage colony stimulating factor (GM-CSF). Consistently, GMSCs enhance skin wound healing by electing polarization of macrophages into the M2 phenotype, indicating that GMSCs prepare a unique microenvironment for tissue repair and remodeling [25]. These findings highlight the immunomodulatory functions of GMSCs on adaptive and innate immune cells and their potential application in cell-based therapy for inflammatory diseases.

### Periodontal ligament stem cells

The periodontal ligament is a connective tissue that connects the cementum to alveolar bone, supporting teeth in the alveolar socket and contributing to tooth nutrition and homeostasis [26]. In 2004, Seo et al. first identified PDLSCs, which can generate a cementum/periodontal ligament-like complex [7]. PDLSCs also exhibit inhibitory effects on PBMC proliferation through suppressing cell division or secreting TGF-β and HGF, but do not induce PBMC apoptosis [21]. In a co-culture system, PDLSCs were shown to induce Treg cells, while suppressing Th17 cell differentiation [27]. Similar to BMMSCs, PDLSCs possess low immunogenicity, and are negative for human leukocyte antigen (HLA)-II DR and co-stimulatory molecules. In the miniature pig periodontitis model, an allogeneic PDLSC sheet was shown to cure periodontitis, perhaps due to low immunogenicity and secretion of PGE2 [3].

### Stem cells from apical papilla

Stem cells from the root apical papilla (SCAP) are isolated from the dental papilla located at the apex of developing human permanent teeth. SCAP can form cell clusters and undergo multilineage differentiation [20]. SCAP possess low immunogenicity and inhibit T-cell proliferation stimulated by PHA in MLRs perhaps via soluble factors [20]. However, the immunological features of SCAP remain elusive.

### Stem cells from exfoliated deciduous teeth

Miura et al. firstly isolated stem cells from human exfoliated deciduous teeth (SHED), a kind of naturally replaced tissue [6]. In addition to their great capacities to proliferate and differentiate into osteogenic cells, adipogenic cells, and odontogenic cells, SHED show remarkable immunosuppressive effects [4]. SHED display profound capacity to inhibit Th17 cell differentiation, which might contribute to the therapeutic effects of SHED on systemic lupus erythematosus (SLE) in mice [4]. More recently, a study showed that systemic infusion of SHED ameliorated the ovariectomy (OVX)-induced osteopenia phenotype in mice by reducing the numbers of Th1 and Th17 cells. Mechanistically, SHED transplantation induces activated T-cell apoptosis via Wnt-β-catenin-Fas/FasL-mediated apoptosis pathways and leads to upregulation of Treg cells and downregulation of Th1 and Th17 cells. Moreover, the immunomodulatory capacity of SHED can be enhanced by acetylsalicylic acid treatment. ASA treatment elevated TERT/FASL signaling in SHED, improving the capacity of SHED-inducing T-cell apoptosis and ameliorating the DSS-induced colitis phenotype in mice [28].

### Stem cells from dental follicle

Stem cells isolated from the dental follicle surrounding the developing tooth show the ability to form colonies, to express differentiated tissue markers, including nestin and Notch1, and to form periodontal ligament-like structures [29]. Stem cells from the dental follicle also suppress the proliferation of PBMCs, which is regulated by Toll-like receptor 4 (TLR4) agonists [29]. Further studies on the immunomodulatory properties of this cell population are still in urgent demand.

### Bone marrow stem cells derived from jaw bone

Akintoye et al. compared bone marrow stem cells isolated from orofacial (maxilla and mandible) and axial (iliac crest) regions and found that orofacial MSCs proliferate more rapidly, have delayed senescence with higher expression level of alkaline phosphatase (ALP), and exhibit more calcium accumulation in vitro [30]. Yamaza et al. isolated and expanded orofacial MSCs from mice and found that these orofacial MSCs showed stronger immunosuppressive effects than BMMSCs by inhibiting T-cell proliferation [8]; orofacial MSCs producing more NO may be one of the contributing factors [8, 30].

These studies reiterate that craniofacial stem cells have a higher immunomodulation capacity than BMMSCs, indicating that they may be excellent cell sources for infectious and inflammatory diseases. However, the mechanism underlying this unique property needs to be further investigated.

## Impact of the inflammatory milieu on craniofacial MSCs

Since Liu et al. reported that recipient T cells impair BMMSC-based tissue engineering via IFN-γ and TNF-α-induced apoptosis, investigation on the interaction of the immune microenvironment and MSCs has made remarkable progress [31]. Given the unique anatomical location of craniofacial MSCs and their higher exposure to the immune stimulus, several studies have implicated distinct interactions between immune cells and craniofacial MSCs. BMMSCs are well known to express a variety of markers that interact with immune system, for instance receptors for cytokines (IL-1, IFN-γ, and TNF-α), TGF-β, and chemokine [32]. Craniofacial MSCs also express these markers to interact with immune cells. Cytokines and growth factors secreted by immune cells modulate the recruitment, proliferation, and differentiation of craniofacial MSCs. For instance, dental stem cell migration is mediated by several cytokines including TGF-β, vascular endothelial growth factor (VEGF), and fibroblast growth factor (FGF)-2. The proliferation and differentiation of craniofacial MSCs can be stimulated by several factors, such as FGF-2, TGF-β, bone morphogenetic protein (BMP)2, and BMP7 [33]. The craniofacial MSC-mediated healing process was also regulated by immune cells by stimulating stem cell migration, proliferation, and differentiation. Conversely, DPSCs isolated from inflamed pulp show lower osteogenic/dentinogenic, adipogenic, and neurogenic differentiation potential than healthy DPSCs, and TNF-α and IL-1β combination treatment decreases the osteogenic/dentinogenic differentiation of DPSCs in vitro.

**Fig. 2:**
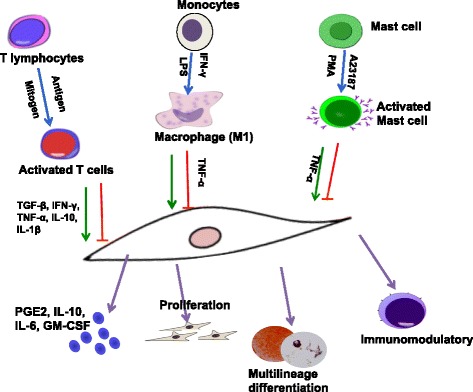
Impact of the immune milieu on craniofacial MSCs. Immune cells secrete inflammatory factors such as interferon gamma (*IFN-γ*), tumor necrosis factor alpha (*TNF-α*), transforming growth factor beta (*TGF-β*), and interleukin-1 beta (*IL-1β*), which regulate craniofacial MSC proliferation, multilineage differentiation, and immunomodulation. Moreover, innate and adaptive immune cells, activated by mitogen or cytokine, secrete inflammatory factors which provide positive or negative feedback to MSCs to regulate a variety of inflammatory factors and their properties. *GM-CSF* granulocyte-macrophage colony stimulating factor, *LPS* lipopolysaccharide, *PGE2* prostaglandin E2

The immunomodulatory capacity of craniofacial MSCs is also regulated by the immune milieu. For instance, the capacity of dental follicle stem cells to suppress PBMC proliferation can be attenuated by TLR4 agonists [29]. Moreover, inflamed PDLSCs show diminished inhibitory effects on T-cell proliferation. In a co-culture system, inflamed PDLSCs exhibit compromised capacity to inhibit Th17 cell differentiation [27]. DPSCs from inflamed pulp are highly dysfunctional in terms of their stemness and immunomodulatory properties, displaying a lower capacity to suppress mitogen-induced T-cell proliferation, lower expression of HLA-ABC, and HLA-G, and higher expression of TNF-α, IL-1β, and IL-2. The immunomodulatory properties of craniofacial MSCs operate via cell-cell contact or through soluble factors. In a co-culture system, the inflammatory cytokines IL-1β, IFN-γ, and TNF-α, which are secreted by activated T lymphocytes and macrophages, have be seen to serve as negative or positive feedback signals in the cross-talk between MSCs and immune cells [22]. These studies indicate that innate and adaptive immune cells influence the regenerative and immunomodulatory properties of craniofacial MSCs via secreting soluble factors (Fig. 2).

## Implications for disease treatment

Craniofacial MSCs, demonstrating a strong capacity for inhibiting proinflammatory processes, have great potential for the treatment of autoimmune disorders such as SLE, osteoporosis, and colitis [9, 34]. Additionally, craniofacial MSCs derived from the neural crest are, in particular, amenable to differentiating into neural cells, indicating that these MSCs could be used to treat disorders of the nervous system, such as Alzheimer's disease, Parkinson's disease, traumatic brain and spinal cord injury, and neuroinflammatory multiple sclerosis [35].

PDLSCs and DPSCs have been reported to repair periodontal bone defects in periodontitis. However, complex ecosystems of commensal bacteria in the oral cavity may trigger an inadequate host inflammatory-immune response, leading to the disruption of tissue homoeostasis and regeneration. It remains a challenge to induce bone regeneration under such harsh environmental conditions. Abe et al. showed that low concentrations of *P. gingivalis* extracts improve osteogenic differentiation of DPSCs, while high concentrations may inhibit ALP activity and bone sialoprotein gene expression [36]. *P. gingivalis* lipopolysaccharide (LPS) was shown to suppress osteoblastic differentiation of DPSCs and PDLSCs. Nomiyama et al. suggested that Gram-negative bacterial infection might downregulate the odontoblastic properties of rat DPSCs after stimulation with *A. actinomycetemcomitans* LPS [37].

Bisphosphonate-related osteoporosis of the jaw (BRONJ), an immune-related disease in the orofacial region, is a side effect of bisphosphonate therapy for cancer or osteoporosis. Systemic infusion of MSCs can cure BRONJ in mice via inhibition of Th17 cells and elevation of Treg cells [38]. These studies indicate that the development of new experimental settings to better imitate the in vivo periodontal milieu seems to be crucial for the potential of MSC-based therapies to be illustrated.

## Conclusions and perspective

The potent immunomodulatory ability of craniofacial MSCs makes them promising candidates to treat autoimmune diseases. Mechanically, craniofacial MSCs interact with the immune stimulus through secreting multiple soluble factors that inhibit immune cell proliferation, inducing immune cell apoptosis via the Fas/FasL pathway, as well as promoting Treg cell differentiation and inhibiting Th17/Th1 cell differentiation.

The challenge posed by the oral environment, with its active immune responses and wide range of bacteria, may contribute to the orofacial MSCs as a distinct population. Craniofacial MSCs may be proper cell resources for treating immune-related diseases in the orofacial regions, such as periodontitis and BRONJ. In return, innate and adaptive immune cells can affect MSC proliferation, differentiation, and even immunomodulation, thereby forming feedback that can attenuate the regenerative and immunomodulatory properties of craniofacial MSCs.

There are several important issues that still need to be addressed before the clinical application of craniofacial MSCs. It needs to be clarified how craniofacial MSCs function in immune-related diseases that affect the orofacial region. In addition, investigation of the interplay between craniofacial MSCs and microorganisms will help to interpret the impact of the microenvironment on MSC properties. Furthermore, how the unique role of tissue-specific immune cells affects the function of craniofacial MSCs in local tissues needs to be addressed. Answers to these issues will substantially enhance the understanding of the properties of craniofacial MSCs and contribute to the future application of the cells.

## Abbreviations

ALP: Alkaline phosphatase
BMMSC: Bone marrow mesenchymal stem cell
BMP: Bone morphogenetic protein
BRONJ: Bisphosphonate-related osteoporosis of the jaw
CFU-F: Colony-forming unit-fibroblasts
CNCC: Cranial neural crest cell
COX2: Cyclooxygenase 2
DC: Dendritic cell
DPSC: Dental pulp stem cell
DSS: Dextran sulfate sodium
FasL: Fas Ligand
FGF: Fibroblast growth factor
GM-CSF: Granulocyte-macrophage colony stimulating factor
GMSC: Mesenchymal stem cell derived from gingiva
HGF: Hepatocyte growth factor
HLA: Human leucocyte antigen
IDO: Indoleamine 2,3-dioxygenase
IFN: Interferon
IL: Interleukin
LPS: Lipopolysaccharide
MLR: Mixed lymphocyte reaction
MSC: Mesenchymal stem cell
NK: Natural killer
NO: Nitric oxide
PBMC: Peripheral blood mononuclear cell
PDLSC: Periodontal ligament stem cell
PGE2: Prostaglandin E2
SCAP: Stem cells from the root apical papilla
SHED: Stem cells of human exfoliated deciduous teeth
SLE: Systemic lupus erythematosus
TGF: Transforming growth factor
TLR4: Toll-like receptor 4
TNF: Tumor necrosis factor
Treg: Regulatory T
TSG-6: Tumor necrosis factor-inducible gene 6
